# Comparative predictive performance of the PopGrouper: internal validation of a population-based classification system for morbidity adjustment using German administrative data

**DOI:** 10.1186/s12913-026-16108-3

**Published:** 2026-11-12

**Authors:** Anika Kreutzberg, Chrissa Tsatsaronis, Wilm Quentin, Reinhard Busse

**Affiliations:** 1 Department of Health Care Management, Technische Universität Berlin, Straße des 17. Juni 135, 10623 Berlin, Germany; 2 Department for Planetary and Public Health, Universität Bayreuth, Universitätsstraße 30, 95447 Bayreuth, Germany

**Keywords:** Morbidity adjustment, Claims data, Multimorbidity, Predictive validity, Population classification, Healthcare costs

## Abstract

**Background:**

To compare the predictive performance of a newly developed population classification system, the PopGrouper, with established morbidity measures used in administrative data research for 1-year mortality, hospitalisation, and healthcare costs.

**Methods:**

This study presents an internal validation using an independent hold-out sample derived from 2022 to 2023 claims data from BARMER, a large German statutory health insurer covering about 10% of the national population (> 8 million). All adults (≥ 18 years) continuously insured in 2022–2023 or deceased in 2023 were included of which a 20%-random sample (*n* = 1,310,273) was used as validation cohort. Regression models were estimated for different outcomes, using age-sex groups, the Charlson Comorbidity Index, Elixhauser comorbidities, PopGroup severity quintiles, Macro PopGroups, or PopGroups as morbidity adjustment methods. For mortality and hospitalisation, sparsely populated PopGroups were hierarchically aggregated to ensure a minimum number of events per group. Predictive performance was assessed using the coefficient of determination (R²) and root mean square error (RMSE) for log-transformed cost outcomes, and c-statistics for mortality and hospitalisation, based on 1,000 bootstrap samples.

**Results:**

The PopGroup model explained 35.8% of the variation in log-transformed total healthcare costs, compared with 11.5% for age-sex groups, 13.2% for Charlson, and 18.6% for Elixhauser. Discrimination for mortality was excellent (c-statistic = 0.884 [95% CI: 0.882–0.886]) and acceptable for hospitalisation (0.714 [0.712–0.715]). Differences compared with conventional morbidity measures were most pronounced for healthcare cost prediction, whereas gains for mortality and hospitalisation discrimination were smaller. Sensitivity analyses yielded consistent findings, confirming robustness across model specifications.

**Conclusions:**

The PopGrouper is an open and reproducible classification system which captures multimorbidity by building clinically and economically meaningful groups derived from administrative data. In this internal validation, it demonstrated strong performance across both clinical and economic outcomes, with the largest improvements observed for healthcare cost prediction. The PopGrouper may therefore provide a useful alternative tool for morbidity adjustment, particularly in applications involving healthcare planning, resource allocation, and population health management.

**Supplementary Information:**

The online version contains supplementary material available at https://doi.org/10.1186/s12913-026-16108-3.

## Background

A large body of literature has been devoted to the development and validation of morbidity measures for morbidity adjustment in health services research. In observational studies, morbidity adjustment is crucial as morbidity may be a potential confounder, effect modifier, or predictor of the outcome of interest [1]. Various morbidity measures are applied in cohort studies using administrative data. Diederichs et al. [2] provide a comprehensive systematic overview. Commonly used measures are the Charlson Comorbidity Index [3] and the Elixhauser comorbidities [4]. Both measures were developed on adult inpatient populations in the United States but have been used across many settings; and several adaptations have been developed for use with different data sources [5]. More recently developed morbidity measures are often validated by comparing their predictive performance to Charlson and Elixhauser. The M3 index based on chronic conditions identified from routine hospital admission data from New Zealand demonstrated improved predictive performance for 1-year mortality risk over Charlson and Elixhauser indices [6]. A morbidity measure based on ICD-10 chapters and groups developed by Stausberg & Hagn [7] using routine data from three German hospitals also performed better than Charlson and Elixhauser in predicting hospital death. Most recently, the Cambridge Multimorbidity Score developed by Payne et al. [8] based on general practitioner electronic record data from the United Kingdom performed significantly better in predicting primary care consultations, unplanned hospital admissions, and death than Charlson.

Another, less frequently discussed, approach to measuring morbidity at a population level is the use of a population classification system based on administrative data. These systems aim to categorise individuals of an entire population into mutually exclusive groups, with each group representing a specific combination of demographic and diagnostic or medical attributes, which offers potential prognostic value for clinically relevant outcomes [9]. The grouping is based on a set of formal classification rules which are either completely data-driven using statistical clustering methods [10] or combine data-driven methods with clinical expertise to build clinically meaningful categories [9]. These systems consider a person’s health conditions as a holistic set, including interactions among multiple health conditions and their impact on healthcare utilisation and mortality across different sectors of care. In contrast to diagnosis-based comorbidity measures such as Charlson or Elixhauser, population classification systems may additionally incorporate information on healthcare utilisation, disease severity, or care needs to characterise individuals with similar expected resource use and health outcomes. Thus, they capture multimorbidity as the co-occurrence of multiple chronic or acute diseases within a single person [11]. With the increase in the prevalence of chronic diseases and demographic changes, multimorbidity poses an increasingly significant challenge to health systems and the delivery of healthcare [12]. Classification systems such as the Adjusted Clinical Groups (ACG) [13] or the Clinical Risk Groups (CRG) [14] are employed in many countries as policy instruments to describe health needs of populations and tailor health services to health needs [15]. Moreover, such systems support a wide range of applications in health services research, including the evaluation of new care models [16] and the analysis of payment model impacts [17]. They have shown strong performance in predicting healthcare expenditure and identifying high-cost patients [18–20].

The PopGrouper is a recently developed population classification system built on claims data in Germany [21]. Its primary aim is to support healthcare needs assessments and resource planning in Germany. Hence, the development of the system optimized its ability to define medically meaningful groups of patients with similar concurrent (same year) costs. However, the PopGrouper may potentially have wider applications in health services research if it can also predict outcomes in subsequent years. This paper aims to evaluate the comparative predictive performance of the PopGrouper with age-sex groups, the Charlson Comorbidity Index and Elixhauser comorbidities which are among the most widely used measures of overall morbidity burden using administrative data [22] for 1-year health outcomes. Because the PopGrouper was developed as a population classification system rather than a diagnosis-based comorbidity measure, differences in predictive performance may reflect both methodological and informational differences between these approaches. Comparing the PopGrouper with established morbidity measures therefore provides insight into the potential added value of a broader representation of multimorbidity for morbidity adjustment.

## Methods

This is a retrospective observational study comparing the predictive performance of the PopGrouper with established morbidity measures for 1-year mortality, hospitalisation, and healthcare costs. Reporting of this study follows the TRIPOD + AI guidance for reporting clinical prediction models that use regression or machine learning methods [23] (see Supplement 1).

### Data and study population

The analysis is based on claims data from one of the largest German statutory health insurance funds, BARMER, covering about 10% of the German population (over 8 million insured persons) nationwide in 2022–2023. The data include individual-level information on demographics and all health and long-term care services covered by statutory health insurance in Germany. The PopGrouper 1.0 was originally developed using a development cohort (80%) and internally validated using an independent hold-out validation cohort (20%). Insured individuals with plausible age (< 125 years) were randomly assigned to the development (*n* = 7,070,986) and validation dataset (*n* = 1,767,155). Claims data were generally checked for plausibility by BARMER before being made available for research purposes. Any additional pre-processing data steps and quality checks are detailed in Braun et al. [21]. The present study was conducted in the validation cohort with additional exclusion criteria applied:

- no continuous insurance with BARMER in 2022–2023, except for individuals who died during 2023 and remained continuously insured until death (development cohort: *n* = 1,071,994 (15%); validation cohort: *n* = 267,285 (15%)).
- age < 18, to ensure sufficient numbers of mortality and hospitalisations (development: *n* = 755,812 (11%); validation: *n* = 189,459 (11%)).
- implausible cost data (cost < 0), as indication for implausible healthcare utilisation records and thus impaired outcome measurement (development: *n* = 585 (< 0.01%); validation: *n* = 141 (< 0.01%)).

### Morbidity measures

#### PopGrouper 1.0

The PopGrouper is a classification algorithm that groups individuals into medically and economically homogeneous categories based on diagnoses and healthcare utilisation within one calendar year. The grouping algorithm follows a hierarchical approach. First, ICD-10 diagnoses are grouped into diagnostic groups and subsequently aggregated into 32 Macro Disease Groups (MDGs) and 433 Consolidated Disease Groups (CDGs), which summarise clinically related conditions across organ systems and medical disciplines. Individuals may be assigned to multiple CDGs, which are characterised by a composite severity score based on standardised mortality ratios, average healthcare costs, and healthcare utilisation. In a second step, individuals are assigned to one of ten Macro PopGroups according to morbidity severity and resource use. Within Macro PopGroups, empirically derived combinations of disease groups are used to construct Base PopGroups, which are further subdivided into PopGroups using individual-level characteristics such as age, sex, level of care dependency, and ventilation requirements. PopGrouper 1.0 was developed on 2022 claims data and comprises 10 Macro PopGroups, 610 Base PopGroups, and 776 PopGroups. In addition, each PopGroup is assigned a composite severity score which compares PopGroups based on medical severity, economic burden, and healthcare utilisation. The severity score ranges between −0.4 and 13.1 with lower values indicating less severe and higher values more severe PopGroups relative to all PopGroups in the classification. The complete technical specification, including all grouping rules, data requirements, and aggregation procedures, has been published separately by Braun et al. [21]. An English-language overview of the grouping logic and hierarchical assignment process is provided by Kreutzberg et al. [24]. In the present study, Macro PopGroups, PopGroups, and PopGroup severity quintiles (very mild to very severe) were used.

#### Age-sex groups

Age was categorised into eight groups (18–24, 25–34, 35–44, 45–54, 55–64, 65–74, 75–84, >=85). Sex was defined as a binary variable (1 = female; 0 = male). Age-sex groups were built as the combination of the two variables.

#### Charlson comorbidity index and Elixhauser comorbidities

The Charlson Comorbidity Index and Elixhauser comorbidities were derived from the same claims data using ICD-10 coding algorithms by Quan et al. [22]. Both measures included all inpatient diagnoses (primary and secondary) as well as confirmed outpatient diagnoses from 2022 to ensure comparability with the PopGrouper. The Charlson Index was based on 17 disease categories (Deyo modification [25]) weighted using the original weights. The Elixhauser measure followed the University of Manitoba SAS macro, comprising 31 comorbidity categories [26]. In the regression models, Charlson was entered as a categorical variable (17 categories for each single score 0–16, and one combined for scores 17–19), and each Elixhauser comorbidity as a separate dummy variable, as recommended by Elixhauser et al. [4].

### Outcome measures

Outcome measures were defined as follows: (1**)** total healthcare costs (all 2023 costs for inpatient and outpatient treatment, medication, rehabilitation, aids, and remedies); (2) inpatient costs (all 2023 inpatient treatment costs); (3) outpatient costs (all 2023 outpatient/ambulatory treatment costs); (4) mortality (1 = deceased in 2023); and (5) hospitalisation (1 = at least one overnight hospital stay in 2023). Mortality and hospitalisation were analysed as binary outcomes within a predefined 1-year follow-up period rather than as time-to-event outcomes. This approach was chosen to facilitate comparison of predictive performance across morbidity measures and is consistent with the validation strategy commonly applied to established morbidity measures such as Charlson and Elixhauser.

### Comparative validation analyses

Correlations between the PopGroup severity score, and Charlson, Elixhauser, as well as Macro PopGroups were analysed using boxplots. Comparative predictive performance was assessed through regression analyses. For each outcome, six regression models were estimated, each including one morbidity measure as the sole predictor (age-sex groups, Charlson, Elixhauser, PopGroup severity quintiles, Macro PopGroups, or PopGroups).

For cost outcomes (total, inpatient, outpatient), general linear models were applied using the natural logarithm of costs as the dependent variable to address right-skewness and meet model assumptions. Predictive performance of the log-transformed cost outcomes was evaluated using the coefficient of determination (R²) and root mean square error (RMSE). R² and RMSE were calculated on the log-cost scale and were used to compare predictive performance across morbidity measures rather than to quantify explained variance in costs measured in euros. Higher R² values and lower RMSE values indicate better model fit.

For mortality and hospitalisation, logistic regressions were estimated. PopGroups with fewer than 100 events were hierarchically aggregated to Base or Macro PopGroups to ensure stable estimation (referred to as “Aggregated PopGroups”). For mortality, MPG P02 (pregnancy, childbirth and postpartum period) was additionally excluded because it contained only four deaths. Because the Aggregated PopGroup models exhibited quasi‑incomplete separation in several groups, these models were estimated with Firth’s bias‑reduced penalised likelihood to obtain stable and unbiased parameter estimates [27].

Discrimination for mortality and hospitalisation was assessed using the c-statistic, equivalent to the area under the receiver operating characteristic (ROC) curve (AUC), with values ranging between 0.5 and 1. The c-statistic reflects the ability of each morbidity measure to discriminate between individuals with and without the outcome. ROC curves were plotted to visualise sensitivity versus 1–specificity.

Each model was run on 1,000 bootstrap samples drawn from the study cohort. Reported estimates represent medians across bootstraps, with 95% confidence intervals derived from the central 95% of the bootstrap distribution. Analyses were conducted using SAS Enterprise Guide 8.3.

### Sensitivity analyses

Several sensitivity analyses were performed. First, persons that died during 2023 were excluded to test any impact on predicting costs and hospitalisation due to these persons having a shorter observation period. Second, 5% of persons with the highest costs were excluded to assess whether the comparative predictive performance was driven by a relatively small number of exceptionally high-cost cases commonly observed in healthcare cost data. These sensitivity analyses used the same methods as described above.

Third, additional models combining age-sex groups with each morbidity measure were estimated to assess the incremental predictive value of the morbidity measures beyond basic demographic characteristics. Fourth, we conducted a high-risk overlap analysis comparing the Aggregated PopGroup and Elixhauser models for mortality and hospitalisation to assess whether the PopGrouper identifies high-risk individuals different from those identified by a simpler diagnosis-based morbidity measure. Individuals were classified as high-risk if they belonged to the top 10% of predicted risk for the respective outcome according to each model. High-risk status was cross-classified between both models, and observed outcome rates as well as mean predicted risks were calculated for concordant and discordant groups. Agreement between the two high-risk classifications was quantified using Cohen’s kappa. Fifth, to provide additional context beyond summary performance measures, we assessed agreement between observed and predicted outcomes across deciles of predicted risk or cost. These analyses were intended to complement c-statistics, R², and RMSE by illustrating agreement between observed and predicted values across the outcome distribution. To limit computational burden, these latter three analyses were conducted in the full validation cohort without bootstrapping.

## Results

### Descriptive results

Comparative descriptive statistics are presented in Table 1. The development cohort included 5,242,605 (80%) and the validation cohort 1,310,273 (20%) persons. The distribution of patient demographics, morbidity measures, and outcomes was similar in both cohorts. Mean age was 57 years (range = 18–110). Over 58% of persons were female. The largest groups in each cohort had a Charlson index of zero (> 52%), one to five Elixhauser comorbidities (> 58%), and a PopGroup in the very mild severity quintile (> 65%). In both cohorts, 4.1% died and 15.5% were hospitalised in 2023. Average total costs per person were 3,394€ (development cohort) and 3,390€ (validation cohort).

**Table 1:** Description and comparison of development and validation cohort data sets

Variable	Level	Development cohort		Validation cohort		
Total, n (%)		5,242,605	(100.0)	1,310,273	(100.0)	
Sex, n (%)	Female	3,063,320	(58.4)	764,746	(58.4)	
	Male or other	2,179,285	(41.6)	545,527	(41.6)	
Age category, n (%)	18–24	253,383	(4.8)	63,236	(4.8)	
	25–34	533,545	(10.2)	133,812	(10.2)	
	35–44	656,828	(12.5)	164,446	(12.6)	
	45–54	734,421	(14.0)	183,390	(14.0)	
	55–64	1,041,964	(19.9)	260,841	(19.9)	
	65–74	915,961	(17.5)	228,674	(17.5)	
	75–84	761,212	(14.5)	190,006	(14.5)	
	>=85	345,291	(6.6)	85,868	(6.6)	
Charlson Comorbidity Index, n (%)	0	2,761,092	(52.7)	691,629	(52.8)	
	1	933,101	(17.8)	232,103	(17.7)	
	2	549,955	(10.5)	137,416	(10.5)	
	>=3	998,457	(19.1)	249,125	(19.0)	
Elixhauser comorbidities, n (%)	0	1,527,393	(29.1)	383,676	(29.3)	
	1–5	3,089,127	(58.9)	770,642	(58.8)	
	6–13	615,130	(11.7)	153,199	(11.7)	
	>=14	10,955	(0.2)	2,756	(0.2)	
PopGroup severity quintile, n (%)	very mild	3,388,812	(64.6)	847,129	(64.7)	
	mild	977,165	(18.6)	243,652	(18.6)	
	moderate	539,962	(10.3)	135,312	(10.3)	
	severe	266,555	(5.1)	66,710	(5.1)	
	very severe	70,111	(1.3)	17,470	(1.3)	
Died in 2023, n (%)		213,600	(4.1)	53,202	(4.1)	
Hospitalised in 2023, n (%)		812,158	(15.5)	202,503	(15.5)	
Total cost in euro per person, mean (SD)		3,394	(9,197)	3,390	(9,271)	
Inpatient cost in euro per person, mean (SD)		1,541	(7,177)	1,539	(7,090)	
Outpatient cost in euro per person, mean (SD)		789	(1,460)	788	(1,448)	

Note: Charlson Comorbidity Index and Elixhauser comorbidities are presented in grouped categories for descriptive purposes only. These categories were not used in the predictive regression models

Due to the age restriction, 9 out of 10 Macro PopGroups and 753 out of 776 PopGroups were present in the study cohort (validation cohort). Table 2 shows prevalence, mean PopGroup severity score, and mean outcomes for the Macro PopGroups. The same information for the most prevalent PopGroups is described in Supplement 2.

**Table 2:** Prevalence (2022) and mean outcomes (2023) of Macro PopGroups (MPG) in the study cohort

MPG	MPG name	Mean severity	Prevalence (2022)	Mean outcomes (2023)				
				Total cost	Inpatient cost	Outpatient cost	Hospitalised	Mortality
P02	Pregnancy, childbirth and postpartum period	-0.02	15,069	2,867 €	1,221 €	721 €	29%	0.03%
P03	Severe high-cost cases	1.51	50,537	10,371 €	5,785 €	1,384 €	35%	40.6%
P04	Actively treated malignant neoplasms	0.70	18,703	10,852 €	4,889 €	1,824 €	35%	15.2%
P05	At least one severe condition	0.14	154,921	6,493 €	2,905 €	1,229 €	26%	8.1%
P06	At least one moderate condition	-0.09	302,811	4,305 €	1,894 €	1,021 €	20%	3.6%
P07	At least one mild condition	-0.20	331,177	2,829 €	1,206 €	828 €	14%	1.2%
P08	At least one very mild condition	-0.30	207,441	1,281 €	505 €	472 €	8%	0.3%
P09	Healthy users	-0.39	164,490	743 €	368 €	242 €	5%	0.5%
P10	Non-users	-0.43	65,124	575 €	406 €	92 €	4%	1.5%

Note: PG01 “Newborns” was excluded from the analysis due to age > = 18. Mean severity represents relative average severity across PopGroups ranging between − 0.4 and 13.1 – with lower values indicating less severe PopGroups and vice versa

The number of persons per PopGroup ranged from 5 to 164,490, with 0 to 3,452 deaths and 0 to 8,435 hospitalisations per group. For the mortality and hospitalisation models, the event-count-based aggregation of PopGroups resulted in 116 aggregated PopGroups for mortality, and 449 for hospitalisation. In the mortality analysis, 15,069 persons (1.15%) from MPG P02 with only 4 deaths were excluded.

**Fig. 1:**
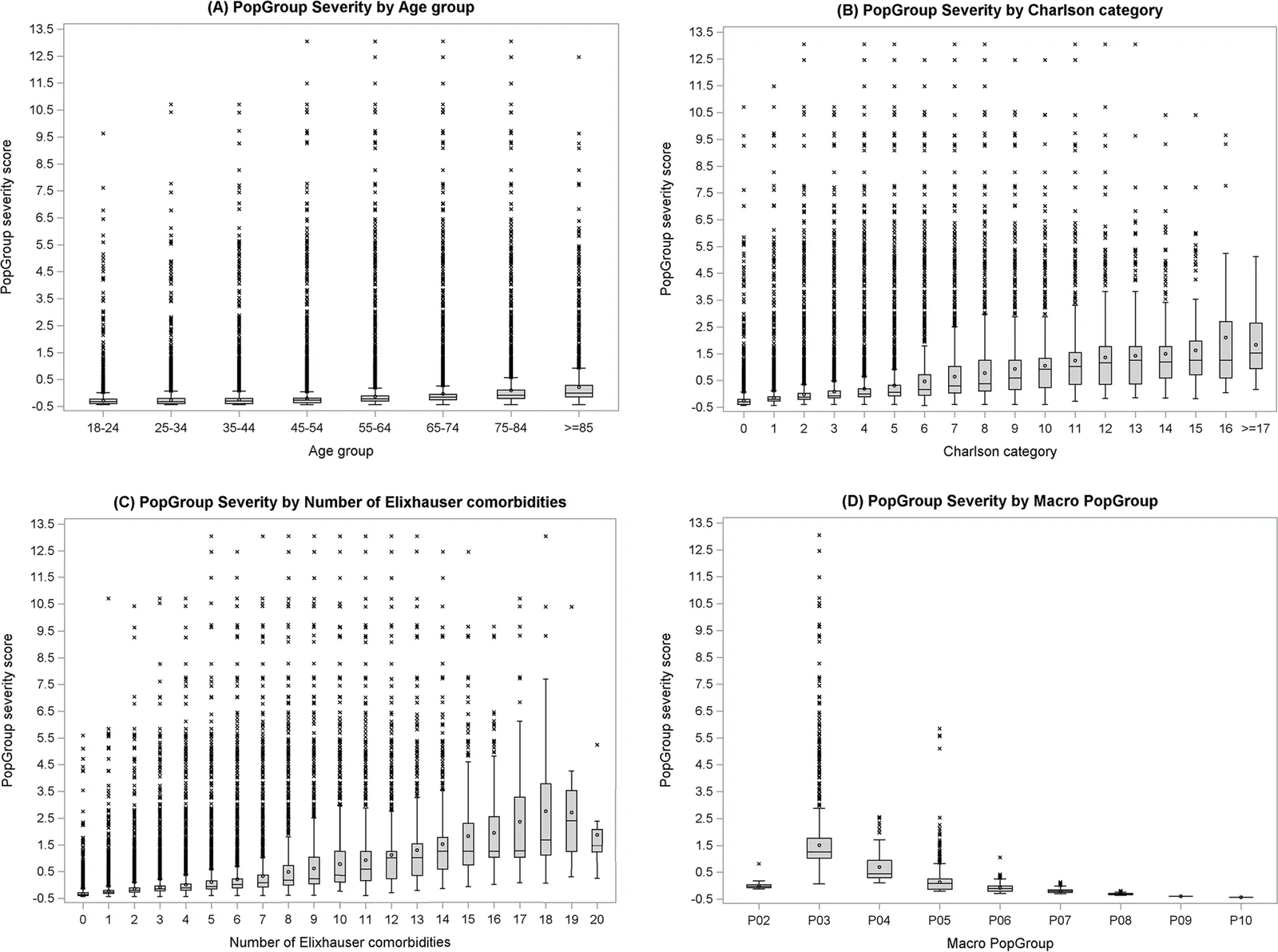
Boxplots of PopGroup severity score by age group (**A**), Charlson category (**B**), Elixhauser comorbidities (**C**), and Macro PopGroups (**D**). Note: PopGroup severity is plotted for all 753 PopGroups present in the study cohort. PopGroup severity represents a standardised composite measure of relative severity across PopGroups ranging from −0.4 to 13.1, with lower values indicating less severe PopGroups and higher values indicating more severe PopGroups relative to all PopGroups in the classification

The boxplots in Fig. 1 visuali​se the distribution of the PopGroup severity score by age group (panel A), Charlson categories (panel B), Elixhauser comorbidities (panel C), and Macro PopGroups (panel D). The boxplots indicate higher average PopGroup severity in the higher age and morbidity groups. At the same time, there is large variation in PopGroup severity by age, Charlson, and Elixhauser categories compared to Macro PopGroups.

### Comparative validation results

Table 3 presents median R² values (with 95%-CI) for predicting total, inpatient, and outpatient costs. Because cost models were estimated using log-transformed costs, reported R² values refer to explained variation on the log-cost scale rather than costs measured in euros. Explanatory power across all morbidity measures was highest for total costs (R²=0.115–0.358), followed by outpatient (0.091–0.355) and inpatient costs (0.052–0.093). The PopGroup model showed the best performance across outcomes, particularly for total and outpatient costs, with substantial gains over age-sex, Charlson, and Elixhauser models. Macro PopGroups also performed well, while PopGroup severity quintiles yielded results comparable to age-sex and Charlson. Differences across models were smallest for inpatient costs. These findings were consistent with RMSE comparisons (Supplement 3). All predictive performance metrics reported were obtained in the independent validation cohort.

**Table 3:** Median R^2^ values and 95% confidence intervals (CI) for predicting 1-year costs (log-transformed) using different morbidity measures

Performance metric: Outcome	Model					
	Age-sex	Charlson	Elixhauser	PopGroup severity	Macro PopGroup	PopGroup
	median [95%-CI]	median [95%-CI]	median [95%-CI]	median [95%-CI]	median [95%-CI]	median [95%-CI]
**R**^**2**^: **Total log-cost**	0.115	0.132	0.186	0.129	0.302	0.358
	[0.114,0.116]	[0.131,0.133]	[0.185,0.187]	[0.128,0.130]	[0.300,0.304]	[0.357,0.360]
Difference from Age-sex				0.014	0.187	0.243
				[0.013,0.016]	[0.185,0.189]	[0.242,0.245]
Difference from Charlson				-0.003	0.17	0.227
				[-0.004,-0.001]	[0.168,0.172]	[0.225,0.228]
Difference from Elixhauser				-0.056	0.116	0.173
				[-0.058,-0.055]	[0.114,0.118]	[0.171,0.174]
**R**^**2**^: **Inpatient log-cost**	0.052	0.067	0.084	0.071	0.06	0.093
	[0.051,0.053]	[0.066,0.068]	[0.083,0.085]	[0.070,0.072]	[0.059,0.060]	[0.092,0.094]
Difference from Age-sex				0.019	0.007	0.041
				[0.018,0.020]	[0.006,0.008]	[0.040,0.042]
Difference from Charlson				0.004	-0.008	0.026
				[0.003,0.004]	[-0.008,-0.007]	[0.025,0.027]
Difference from Elixhauser				-0.014	-0.025	0.009
				[-0.015,-0.013]	[-0.026,-0.024]	[0.008,0.010]
**R**^**2**^: **Outpatient log-cost**	0.098	0.095	0.156	0.091	0.309	0.355
	[0.097,0.099]	[0.094,0.096]	[0.155,0.157]	[0.090,0.092]	[0.307,0.311]	[0.353,0.356]
Difference from Age-sex				-0.007	0.211	0.256
				[-0.009,-0.006]	[0.209,0.213]	[0.254,0.258]
Difference from Charlson				-0.004	0.215	0.26
				[-0.005,-0.003]	[0.213,0.216]	[0.258,0.262]
Difference from Elixhauser				-0.065	0.153	0.198
				[-0.066,-0.064]	[0.151,0.155]	[0.196,0.200]

Note: Estimates represent medians across 1,000 bootstraps, with 95% confidence intervals (CI) derived from the central 95% of the bootstrap distribution. Reported R^2^ values refer to explained variation on the log-cost scale. PopGroup results are based on all 753 PopGroups present in the study cohort

**Fig. 2:**
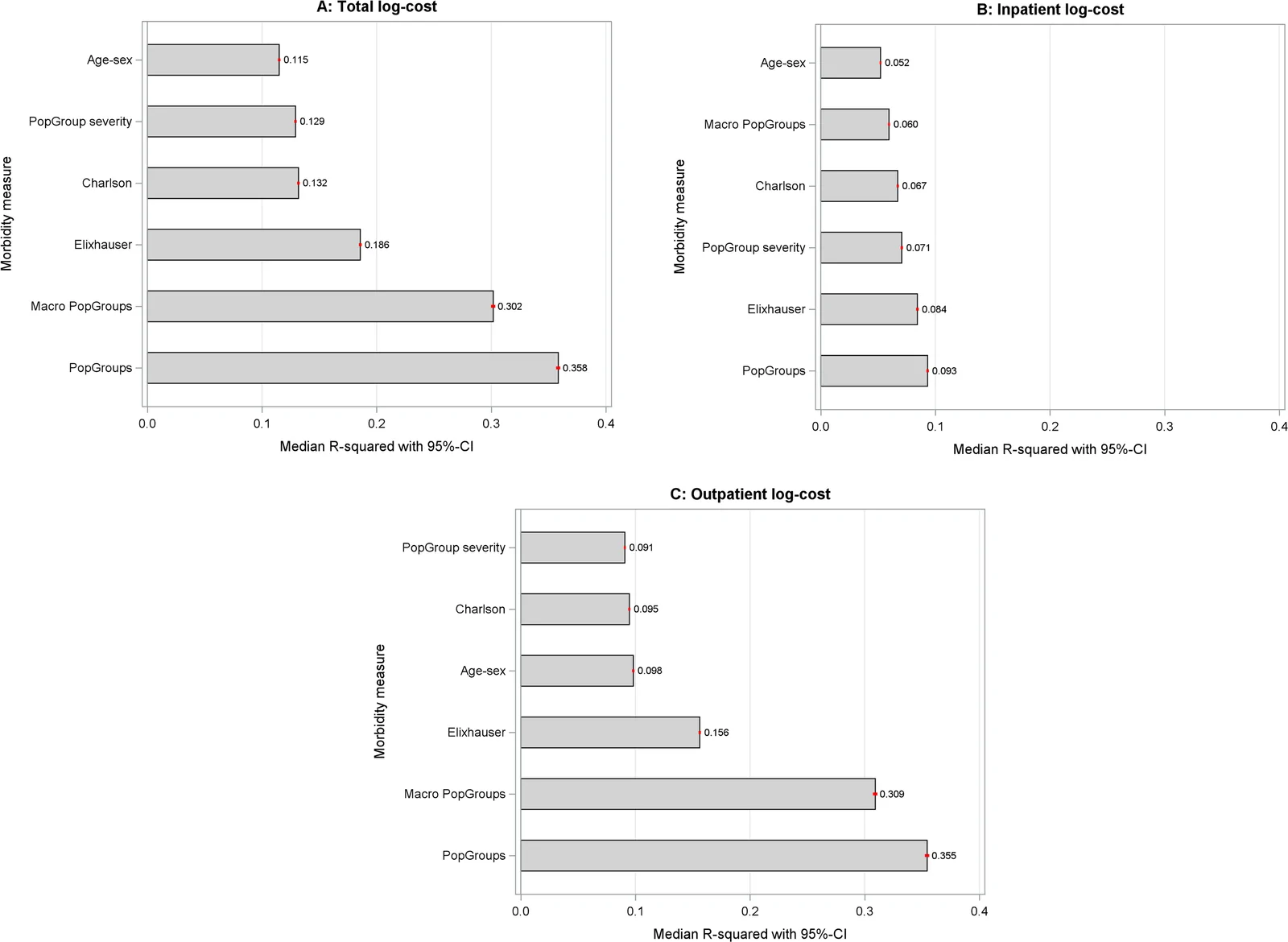
Bar chart of median R^2^ with 95%-confidence interval (CI) for prediction of 1-year cost outcomes (log-transformed). Note: Estimates represent medians across 1,000 bootstraps, with 95% confidence intervals (CI) derived from the central 95% of the bootstrap distribution. Sorted by size of median R^2^. Due to the large sample size, confidence intervals are extremely narrow and may not be fully visible; exact values are reported in Table 3

Figure 2 visualises the median R² values, confirming the superior explanatory power of PopGroups – especially for total and outpatient costs – while inpatient cost models showed uniformly lower R² values.

Table 4 presents median c-statistics (95% CI) for predicting mortality and hospitalisation. For mortality, all models achieved excellent discrimination (c-statistics > 0.8). The Aggregated PopGroup model reached the highest value (0.884 [0.882–0.886]), with a difference in c-statistics of 0.021 to the age-sex, 0.043 to the Charlson, and 0.008 to the Elixhauser model. For hospitalisation, discrimination was lower overall (c-statistics 0.667–0.714), but still within an acceptable range. Again, the Aggregated PopGroup model yielded the highest c-statistic with gains between 0.014 and 0.043 relative to age-sex, Charlson, and Elixhauser. Macro PopGroups and PopGroup severity quintiles yielded results similar to age-sex and Charlson.

**Table 4:** Median c-statistic and 95% confidence intervals for predicting 1-year mortality and hospitalisation by morbidity measure

Performance metric: Outcome	Model					
	Age-sex	Charlson	Elixhauser	PopGroup severity	Macro PopGroup	Aggregated PopGroup
	median [95%-CI]	median [95%-CI]	median [95%-CI]	median [95%-CI]	median [95%-CI]	median [95%-CI]
**c-statistic: Mortality**	0.862	0.841	0.876	0.851	0.844	0.884
	[0.861,0.864]	[0.837,0.843]	[0.874,0.878]	[0.849,0.853]	[0.842,0.846]	[0.882,0.886]
Difference from Age-sex				-0.012	-0.019	0.021
				[-0.014,-0.009]	[-0.021,-0.016]	[0.019,0.024]
Difference from Charlson				0.010	0.003	0.043
				[0.008,0.013]	[0.001,0.007]	[0.041,0.047]
Difference from Elixhauser				-0.025	-0.032	0.008
				[-0.027,-0.024]	[-0.034,-0.031]	[0.006,0.010]
**c-statistic: Hospitalisation**	0.671	0.671	0.700	0.667	0.676	0.714
	[0.670,0.672]	[0.670,0.672]	[0.699,0.701]	[0.666,0.668]	[0.674,0.677]	[0.712,0.715]
Difference from Age-sex				-0.004	0.005	0.043
				[-0.006,-0.003]	[0.003,0.006]	[0.041,0.044]
Difference from Charlson				-0.004	0.005	0.043
				[-0.005,-0.003]	[0.003,0.006]	[0.042,0.044]
Difference from Elixhauser				-0.033	-0.024	0.014
				[-0.034,-0.032]	[-0.026,-0.023]	[0.013,0.015]

Note: Estimates represent medians across 1,000 bootstraps, with 95% confidence intervals (CI) derived from the central 95% of the bootstrap distribution. Aggregated PopGroups represent PopGroups or higher-level Base or Macro PopGroups with a minimum of 100 events per group (116 aggregated PopGroups for mortality and 449 for hospitalisation). For mortality, 15,069 individuals (MPG *P02*) were excluded

**Fig. 3:**
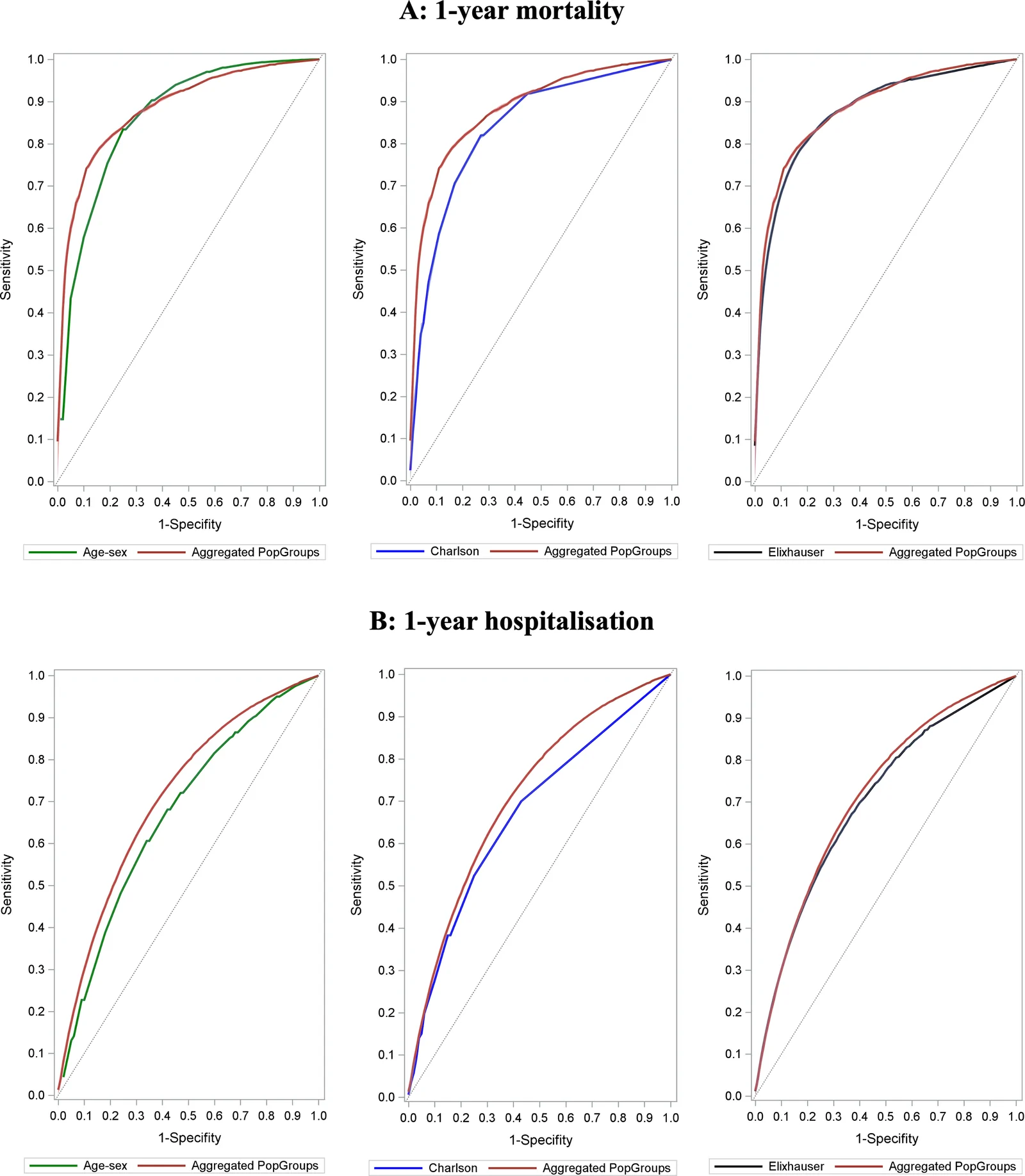
ROC curves by morbidity measure for predicting 1-year mortality and hospitalisation. Note: Estimates represent medians across 1,000 bootstraps, with 95% confidence intervals (CI) derived from the central 95% of the bootstrap distribution. Aggregated PopGroups represent PopGroups or higher-level Base or Macro PopGroups with a minimum of 100 events per group (116 aggregated PopGroups for mortality and 449 for hospitalisation). For mortality, 15,069 individuals (MPG *P02*) were excluded

Figure 3 illustrates these results using ROC curves. For mortality (panel A), all models performed better than random, with the PopGroup model demonstrating very similar sensitivity to Elixhauser and higher sensitivity across most specificity levels compared to Charlson. For hospitalisation (panel B), all models showed lower discrimination, though the PopGroup model performed best, followed by Elixhauser.

### Results of sensitivity analyses

Excluding 4.1% of individuals who died in 2023 (*n* = 53,202) produced similar results for cost and hospitalisation prediction (Supplement 4). PopGroups remained the best-performing measure, with even higher explained variance for total costs (R²=0.449). After excluding the top 5% of high-cost cases (*n* = 65,514), mean total cost decreased to 1,930 euros per person (31,122 euros among excluded cases), yet results remained consistent (Supplement 5). R² values slightly declined due to lower cost variance, but the relative ranking of models was unchanged. PopGroups and Macro PopGroups continued to show the highest explanatory power for total and outpatient costs, with minor differences for inpatient costs. Including age-sex groups alongside each morbidity measure yielded similar results (Supplement 6). Although overall model performance increased, particularly for mortality prediction, the relative ranking of the morbidity measures remained unchanged. These findings confirm the robustness of results across model specifications. Cross-classification of individuals in the top 10% predicted mortality and hospitalisation risk according to the Aggregated PopGroup and Elixhauser models demonstrated only moderate agreement (Cohen’s κ = 0.50 for mortality and κ = 0.45 for hospitalisation; Supplement 7). For mortality, both models identified a common group with very high observed mortality (38.7%), while observed mortality among individuals uniquely classified as high-risk was 14.0% for Aggregated PopGroups and 10.3% for Elixhauser. For hospitalisation, the corresponding observed rates were 30.8% and 31.9%, respectively, compared with 42.4% among individuals classified as high-risk by both models. Agreement analyses comparing observed and predicted values across deciles of predicted risk or cost generally showed close agreement for both the Elixhauser and PopGroup models (Supplement 8). For mortality and hospitalisation, observed event rates closely tracked predicted risks across the risk distribution. For cost outcomes, agreement was particularly close for the PopGrouper, especially for total and outpatient costs, whereas differences between the two approaches were smaller for inpatient costs. These findings are consistent with the observed differences in c-statistics, R², and RMSE.

## Discussion

This internal validation study compared a newly developed population classification system (PopGrouper) with commonly used morbidity measures derived from administrative data and found that PopGroups provided the highest predictive performance across the evaluated outcomes. For the binary outcomes, this comparison was based on discriminatory performance as measured by the c-statistic. Across all sensitivity analyses the relative ranking of the morbidity measures remained unchanged, supporting the robustness of the findings.

For cost predictions, the PopGrouper demonstrated substantial differences in R^2^ compared to age-sex adjustment and traditional comorbidity indices. Both PopGroups and Macro PopGroups reached an R^2^ above 0.30 for predicting total and outpatient cost, which is higher than the range typically reported for claims-based risk adjustment models [28] and in line with findings from prior studies on the predictive performance of population classification systems [18–20]. Similar to findings from Caballer-Tarazona et al. [18], explanatory power for inpatient costs was lower. Inpatient costs are episodic and driven by relatively rare, high-severity events, which compress R^2^ across all models despite small but consistent improvements for PopGroups compared to the other models. The stronger performance of the PopGrouper for cost prediction should be interpreted in light of its design. Unlike Charlson and Elixhauser, which are diagnosis-based comorbidity measures, the PopGrouper incorporates information on healthcare utilisation as part of the classification process. Because healthcare utilisation is closely related to subsequent healthcare expenditure, some improvement in cost prediction compared with diagnosis-only measures is expected. The comparison therefore reflects differences between alternative approaches to morbidity adjustment rather than a comparison of measures based on identical inputs. Supplementary agreement analyses comparing observed and predicted mean log-costs across deciles of predicted cost further illustrated these differences. For total and outpatient costs, the PopGrouper showed substantially closer agreement between observed and predicted costs across the cost distribution than the Elixhauser model, whereas differences were smaller for inpatient costs. These findings are consistent with the observed differences in R² and suggest that the superior cost prediction achieved by the PopGrouper is not limited to a specific segment of the cost distribution.

For mortality, the PopGrouper’s discriminative ability was similar to age-sex, Charlson and Elixhauser models with c-statistics ranging between 0.84 and 0.88. These findings indicate strong discrimination between individuals who did and did not experience the outcome. Agreement analyses comparing observed and predicted event rates across risk deciles also showed generally close agreement for both the Elixhauser and Aggregated PopGroup models, with slightly closer agreement for the PopGrouper. For hospitalisation, discriminative ability was lower across all models, consistent with the more complex and multifactorial determinants of hospital admission (e.g., local supply, practice patterns, social and functional needs) compared with death. Nevertheless, PopGroups achieved the highest discrimination. Additional risk-stratification analyses suggested that the modest differences in discrimination between the Aggregated PopGroup and Elixhauser models do not simply reflect minor changes in the ranking of the same individuals. Although both approaches identified a common group of individuals at very high risk, agreement between the high-risk groups was only moderate. A substantial proportion of individuals were classified as high-risk by only one of the two models, indicating that the PopGrouper captures risk profiles that are not fully represented by diagnosis-based comorbidity measures alone. This observation suggests that the additional complexity of the PopGrouper may influence the identification and targeting of high-risk populations, which could be relevant for care management and resource planning applications.

The findings provide insight into when a population classification system may offer advantages over traditional comorbidity measures. Charlson and Elixhauser primarily summarize the presence of diagnoses and have been widely used for morbidity adjustment and outcome prediction. In contrast, the PopGrouper was designed to classify entire populations into clinically and economically meaningful groups that reflect combinations of conditions, disease severity, and healthcare utilisation patterns. This broader representation of multimorbidity may explain why the largest performance gains were observed for healthcare cost prediction, whereas differences in mortality and hospitalisation discrimination were comparatively modest.

From a practical perspective, the choice of morbidity measure should depend on the intended application. For studies primarily concerned with mortality adjustment, established measures such as Elixhauser may provide a parsimonious and well-validated solution. However, for applications involving healthcare planning, resource allocation, population segmentation, risk-adjusted payment systems, or the identification of populations with complex healthcare needs, the PopGrouper may offer additional value by capturing clinically meaningful patient profiles beyond diagnosis counts alone.

### Strengths and weaknesses

The strengths of the PopGrouper lie in its ability to classify every person into mutually exclusive, medically interpretable groups that jointly reflect combinations of medical conditions, severity, and resource use. Unlike additive comorbidity counts or single-score indices, this grouping captures interactions among co-occurring conditions and considers information about care intensity (e.g., outpatient visits, inpatient days). The availability of multiple aggregation levels (Macro PopGroups, Base PopGroups, PopGroups) may further help balance model granularity and statistical stability (via aggregation when events are sparse), while preserving clinically meaningful distinctions. The PopGrouper utilises administrative data which brings the advantage of large population-based samples capturing real-world conditions and minimising selection bias [22]. Diagnostic information is based on claimed diagnoses rather than patient self-report or medical notes review and covers all sectors of healthcare utilisation (inpatient, outpatient, rehabilitative). The grouping rules are published and versioned for replicability. The system can be adapted to more recent data to capture changes in morbidity prevalence, treatment options, and related healthcare utilisation. Over time, there may be conditions with decreasing impact on mortality due to treatment improvements and medical advances, while other conditions will have growing impact on either mortality or healthcare utilisation.

Some limitations need to be considered when interpreting results. First, the findings are based on an internal validation using data from a single German statutory health insurer from which the PopGrouper was also developed. While the use of an independent hold-out sample strengthens the internal validity of the findings, external validity to other populations and settings remains to be established. Second, claims data lack certain clinical, functional, and social determinants that influence costs and utilisation. Misclassification from coding practices is possible [29, 30]. Third, each morbidity measure was used as the sole predictor to provide a clean comparative validation. So reported metrics reflect incremental predictive value attributable to the morbidity measure rather than an optimised multivariable specification. Sensitivity analysis combining age-sex groups with each morbidity measure yielded similar conclusions, indicating that the relative performance of the morbidity measures was robust to adjustment for basic demographic characteristics. Fourth, the differentiated grouping system challenges statistical efficiency in regression analyses particularly when predicting rare events such as mortality. A conservative approach was used by aggregating PopGroups to larger Base or Macro PopGroups to a minimum of 100 events per group which exceeds the commonly cited threshold of 10 events per estimated parameter [31]. Still, residual overfitting cannot be fully excluded despite bootstrap validation. Fifth, mortality was analysed as a fixed 1-year binary outcome using logistic regression. Although appropriate for the study objective and the predefined follow-up period, this approach does not account for differences in the timing of death, and the 1-year horizon may understate longer-term prognostic differences. Future studies could therefore apply survival-analysis methods, such as Cox proportional hazards models, and assess predictive performance over extended follow-up periods. Finally, we did not formally evaluate calibration using measures such as calibration intercepts, slopes, or calibration curves. However, supplementary agreement analyses comparing observed and predicted outcomes across deciles of predicted risk or cost suggested generally close agreement between observed and predicted values. Future studies could extend these analyses using formal calibration metrics, particularly if the PopGrouper were to be applied for individual risk prediction or risk stratification.

### Future research directions

Future work should prioritise external validation using data from other insurers, regions, and healthcare systems. Stratification by subgroups may provide further insights into the heterogeneity of the PopGrouper’s predictive performance. Combining PopGroups with additional covariates (e.g., age, sex, socioeconomic factors) and incorporating multi-year morbidity trajectories may further enhance prediction. Considering the PopGrouper’s primary aim to describe meaningful groups of patients with similar healthcare utilisation within the same year, predictive variant(s) of the PopGrouper could be developed, similar to predictive model variants of ACGs [32], to further improve the system’s ability of predicting costs or health outcomes in subsequent years.

## Conclusion

In this internal validation study, the PopGrouper demonstrated strong performance across a range of healthcare costs and clinical outcomes. Compared with commonly used morbidity adjustment measures, improvements were most pronounced for healthcare cost prediction, while discrimination for mortality and hospitalisation was comparable to or modestly better than existing approaches.

The practical value of the PopGrouper may be greatest in applications that require population segmentation and healthcare resource planning, where capturing combinations of conditions, severity, and healthcare utilisation is particularly relevant. The observed differences in high-risk classification compared with conventional comorbidity measures further suggest that the PopGrouper identifies partially distinct patient populations, which may support targeting of care management and integrated care interventions. As a transparent and reproducible classification system based on clinically and economically meaningful categories, the PopGrouper may provide a useful alternative tool for morbidity adjustment in health services research, healthcare planning, and policy applications.

## Supplementary Information

Below is the link to the electronic supplementary material.

Supplementary Material 1: TRIPOD Checklist

Supplementary Material 2: Most frequent PopGroups

Supplementary Material 3: Median RMSE values for predicting 1-year costs using different morbidity measures

Supplementary Material 4: Results of sensitivity analysis 1: Exclusion of persons deceased in 2023

Supplementary Material 5: Results of sensitivity analysis 2: Exclusion of 5% highest cost persons

Supplementary Material 6: Results of sensitivity analysis 3: Addition of age-sex groups to morbidity measure models

Supplementary Material 7: Results of sensitivity analysis 4: Cross-classification of individuals in the top 10% predicted risk according to the Aggregated PopGroup and Elixhauser models

Supplementary Material 8: Results of sensitivity analysis 5: Agreement between observed and predicted outcomes

## Data Availability

The data utilised are health insurance claims data provided by the German statutory health insurance fund BARMER. Due to legal and privacy restrictions, these data are not publicly available. Access to the data is subject to approval by BARMER and may require additional agreements regarding data protection and confidentiality. The grouping algorithm to build the PopGrouper Version 1.0 is published at the institutional repository of Technische Universität Berlin [21].

## Declarations

### Study registration and public involvement

The study was not registered or protocolled prospectively. During the development of the PopGrouper different stakeholders from health ministries and provider associations were consulted to provide feedback.

## Abbreviations

ACG: Adjusted Clinical Groups
AUC: Area Under the Curve
BARMER: German statutory health insurance fund
CDG(s): Consolidated Disease Group(s)
CI: Confidence Interval
CRG: Clinical Risk Groups
ICD-10: International Classification of Diseases, 10th Revision
R^2^: Coefficient of Determination
RMSE: Root Mean Square Error
ROC: Receiver Operating Characteristic
SAS: Statistical Analysis System
SD: Standard Deviation
