# Supplementary material for: Comparative predictive performance of the PopGrouper: internal validation of a population-based classification system for morbidity adjustment using German administrative data
Source: BMC Health Serv Res. 2026 Nov 12;26:1611. doi: 10.1186/s12913-026-16108-3 (PMC13770333; doi:10.1186/s12913-026-16108-3)
Supplement: Supplementary file 2 [file 12913_2026_16108_MOESM2_ESM.pdf]

Supplement B. Most frequent PopGroups with severity score, prevalence (2022), and mean outcomes (2023) in study cohort (sorted by prevalence)

PG	PG Name	PopGroup severity	Prevalence (2022)	**Mean outcomes** (2023)				
				**Total cost**	**Inpatient cost**	**Outpatient cost**	**Hospi-talized**	**Mortality**
P09001ZZ	P09: Healthy users	-0.39	164,490	743 €	368 €	242 €	5%	0.5%
P10001ZZ	P10: Non-users	-0.43	65,124	575 €	406 €	92 €	4%	1.5%
P05106BB	P05: Other (very) severe condition(s) + at most 13 MDGs without long-term insulin treatment	-0.20	36,713	2,084 €	736 €	713 €	11%	0.8%
P07065BB	P07: Gastrointestinal diseases (MDG) + at most 6 MDGs and age < 80 years	-0.27	27,281	1,504 €	592 €	571 €	10%	0.2%
P08033ZZ	P08: Other very mild condition(s)	-0.35	26,725	736 €	286 €	303 €	5%	0.1%
P06045BB	P06: Diabetes mellitus with disease symptoms affecting at least one organ system + at most 14 MDGs and level of care dependency ≤ 2	-0.07	25,027	5,188 €	2,147 €	1,137 €	21%	2.5%
P07060BB	P07: Mild or moderate depression + at most 6 MDGs and age ≥ 25 years	-0.25	20,010	1,957 €	720 €	826 €	9%	0.4%
P07053BB	P07: Diabetes mellitus without complications + at most 7 MDGs and age ≥ 30 years	-0.23	19,254	2,672 €	1,164 €	714 €	13%	1.7%
P06063BB	P06: Diseases of the lungs (MDG) & Diseases of the heart (MDG) + at most 12 MDGs and level of care dependency ≤ 2	-0.14	18,703	4,425 €	2,187 €	995 €	22%	2.7%
P06087BB	P06: Other moderate condition(s) + at most 9 MDGs and age < 80 years	-0.28	18,224	1,655 €	598 €	609 €	9%	0.2%
P07010BB	P07: Arthritis + at most 11 MDGs and level of care dependency ≤ 1	-0.10	15,437	4,403 €	1,324 €	958 €	16%	0.7%
P07068BB	P07: Other mild condition(s) + at most 4 MDGs and age ≥ 70 years	-0.30	15,404	1,228 €	467 €	446 €	7%	0.2%
P08028ZZ	P08: Metabolic and endocrine diseases (MDG)	-0.31	15,195	1,152 €	434 €	491 €	7%	0.3%
P06023BB	P06: Severe depression + at most 14 MDGs and age ≥ 18 years	0.20	14,714	5,312 €	2,580 €	1,411 €	22%	2.5%
P07040BB	P07: Coronary sclerosis or other chronic ischemic heart disease + at most 8 MDGs and level of care dependency ≤ 1	-0.20	14,353	3,119 €	1,482 €	852 €	18%	1.4%
P08032ZZ	P08: Gynaecological and reproductive diseases (MDG)	-0.32	13,991	891 €	311 €	417 €	6%	0.0%
P06078BB	P06: Injuries (MDG) + at most 10 MDGs and level of care dependency ≤ 1	-0.20	13,544	2,242 €	952 €	722 €	12%	0.4%
P07042BB	P07: Chronic pain + at most 9 MDGs and level of care dependency ≤ 1	-0.20	13,470	2,664 €	1,119 €	850 €	14%	0.8%
P08029ZZ	P08: Diseases of the heart (MDG)	-0.33	13,270	1,373 €	658 €	433 €	8%	1.0%

Note: MDG = Major Disease Group, CDG = Consolidated Disease Group. PopGroup severity represents a standardized composite measure of relative severity across PopGroups, combining medical severity (standardized mortality ratio), economic burden (average total costs), and healthcare utilization (average outpatient visits and inpatient days). The score ranges from -0.4 to 13.1, with lower values indicating less severe PopGroups and higher values indicating more severe PopGroups relative to all PopGroups in the classification. For further explanation of classification rules refer to Braun et al. (2025).
