# Supplementary material for: Comparative predictive performance of the PopGrouper: internal validation of a population-based classification system for morbidity adjustment using German administrative data
Source: BMC Health Serv Res. 2026 Nov 12;26:1611. doi: 10.1186/s12913-026-16108-3 (PMC13770333; doi:10.1186/s12913-026-16108-3)
Supplement: Supplementary file 3 [file 12913_2026_16108_MOESM3_ESM.pdf]

Supplement C. Median RMSE values for predicting 1-year cost outcomes (log-transformed) using different morbidity measures

Performance metric: Outcome	Model					
	**Age-sex groups**<br>median<br>[95%-CI]	**Charlson**<br>median<br>[95%-CI]	**Elixhauser**<br>median<br>[95%-CI]	**PopGroup severity**<br>median<br>[95%-CI]	**Macro PopGroup**<br>median<br>[95%-CI]	**PopGroup**<br>median<br>[95%-CI]
**RMSE: Total cost**	2.33<br>[2.326,2.333]	2.308<br>[2.303,2.311]	2.235<br>[2.230,2.239]	2.311<br>[2.307,2.315]	2.069<br>[2.065,2.073]	1.984<br>[1.980,1.988]
**RMSE: Inpatient cost**	3.132<br>[3.127,3.137]	3.107<br>[3.102,3.112]	3.079<br>[3.074,3.084]	3.101<br>[3.096,3.106]	3.12<br>[3.115,3.125]	3.064<br>[3.059,3.069]
**RMSE: Outpatient cost**	1.965<br>[1.961,1.968]	1.968<br>[1.964,1.972]	1.901<br>[1.897,1.904]	1.973<br>[1.969,1.976]	1.72<br>[1.716,1.723]	1.663<br>[1.659,1.666]

Note: RMSE: root mean square error. CI: Confidence interval. Estimates represent medians across 1,000 bootstraps, with 95% confidence intervals (CI) derived from the central 95% of the bootstrap distribution. RMSE values are reported on the log-cost scale and do not represent prediction error in euros. PopGroup results are based on all 753 PopGroups present in the study cohort.
