# Supplementary material for: Comparative predictive performance of the PopGrouper: internal validation of a population-based classification system for morbidity adjustment using German administrative data
Source: BMC Health Serv Res. 2026 Nov 12;26:1611. doi: 10.1186/s12913-026-16108-3 (PMC13770333; doi:10.1186/s12913-026-16108-3)
Supplement: Supplementary file 4 [file 12913_2026_16108_MOESM4_ESM.pdf]

Supplement D. Results of sensitivity analysis 1: Exclusion of persons deceased in 2023

Performance metric: Outcome	Model					
	**Age-sex groups** median [95%-CI]	**Charlson** median [95%-CI]	**Elixhauser** median [95%-CI]	**PopGroup severity** median [95%-CI]	**Macro PopGroup** median [95%-CI]	**PopGroup** median [95%-CI]
**R^2: Total log-cost**	0.171 [0.170,0.172]	0.209 [0.208,0.210]	0.275 [0.274,0.276]	0.196 [0.195,0.197]	0.392 [0.390,0.394]	0.449 [0.447,0.450]
Difference from Age-sex				0.025 [0.024,0.027]	0.221 [0.218,0.223]	0.277 [0.275,0.280]
Difference from Charlson				-0.013 [-0.014,-0.012]	0.182 [0.180,0.184]	0.239 [0.237,0.241]
Difference from Elixhauser				-0.079 [-0.080,-0.078]	0.117 [0.115,0.118]	0.174 [0.172,0.175]
**R^2: Inpatient log-cost**	0.048 [0.047,0.049]	0.065 [0.064,0.066]	0.089 [0.088,0.090]	0.074 [0.073,0.076]	0.063 [0.062,0.064]	0.095 [0.094,0.096]
Difference from Age-sex				0.027 [0.026,0.028]	0.015 [0.014,0.016]	0.047 [0.046,0.049]
Difference from Charlson				0.009 [0.008,0.010]	-0.003 [-0.004,-0.002]	0.03 [0.029,0.031]
Difference from Elixhauser				-0.014 [-0.016,-0.014]	-0.026 [-0.027,-0.025]	0.006 [0.005,0.007]
**R^2: Outpatient log-cost**	0.152 [0.151,0.153]	0.166 [0.165,0.166]	0.227 [0.227,0.228]	0.137 [0.136,0.138]	0.391 [0.389,0.393]	0.429 [0.427,0.431]
Difference from Age-sex				-0.015 [-0.016,-0.014]	0.239 [0.237,0.242]	0.277 [0.275,0.280]
Difference from Charlson				-0.029 [-0.030,-0.028]	0.226 [0.224,0.228]	0.264 [0.262,0.265]
Difference from Elixhauser				-0.091 [-0.091,-0.090]	0.164 [0.162,0.166]	0.202 [0.200,0.204]
**c-statistic: Hospitalisation**	0.666 [0.664,0.667]	0.666 [0.664,0.667]	0.700 [0.698,0.701]	0.665 [0.664,0.666]	0.676 [0.675,0.678]	0.677 [0.677,0.677]
Difference from Age-sex				0.000 [-0.002,0.001]	0.011 [0.009,0.012]	0.012 [0.010,0.013]
Difference from Charlson				0.000 [-0.002,0.001]	0.011 [0.009,0.012]	0.012 [0.010,0.013]
Difference from Elixhauser				-0.034 [-0.036,-0.033]	-0.023 [-0.025,-0.022]	-0.023 [-0.024,-0.021]

Note: Estimates represent medians across 1,000 bootstraps, with 95% confidence intervals (CI) derived from the central 95% of the bootstrap distribution. Reported R^2 values refer to explained variation on the log-cost scale. For cost outcomes, PopGroup results are based on all 753 PopGroups present in the study cohort. For hospitalization, PopGroup results refer to aggregated PopGroups which represent PopGroups or higher-level Base or Macro PopGroups with a minimum of 100 events per group (449 aggregated PopGroups for hospitalisation).
