# Supplementary material for: Comparative predictive performance of the PopGrouper: internal validation of a population-based classification system for morbidity adjustment using German administrative data
Source: BMC Health Serv Res. 2026 Nov 12;26:1611. doi: 10.1186/s12913-026-16108-3 (PMC13770333; doi:10.1186/s12913-026-16108-3)
Supplement: Supplementary file 5 [file 12913_2026_16108_MOESM5_ESM.pdf]

Supplement E. Results of sensitivity analysis 2: Exclusion of 5% highest cost persons

Performance metric: Outcome	Model					
	**Age-sex groups**	**Charlson**	**Elixhauser**	**PopGroup severity**	**Macro PopGroup**	**PopGroup**
	median [95%-CI]	median [95%-CI]	median [95%-CI]	median [95%-CI]	median [95%-CI]	median [95%-CI]
**R^2: Total log-cost**	0.103 [0.102,0.104]	0.113 [0.111,0.114]	0.173 [0.172,0.174]	0.12 [0.119,0.121]	0.313 [0.311,0.315]	0.368 [0.366,0.370]
Difference from Age-sex				0.017 [0.015,0.018]	0.210 [0.208,0.212]	0.264 [0.262,0.267]
Difference from Charlson				0.008 [0.006,0.009]	0.201 [0.199,0.203]	0.255 [0.253,0.257]
Difference from Elixhauser				-0.053 [-0.054,-0.052]	0.140 [0.138,0.142]	0.194 [0.192,0.196]
**R^2: Inpatient log-cost**	0.029 [0.029,0.030]	0.031 [0.030,0.032]	0.04 [0.040,0.041]	0.034 [0.033,0.034]	0.032 [0.031,0.032]	0.051 [0.050,0.052]
Difference from Age-sex				0.004 [0.004,0.005]	0.002 [0.002,0.003]	0.022 [0.021,0.023]
Difference from Charlson				0.003 [0.002,0.003]	0.001 [-0.000,0.001]	0.020 [0.019,0.021]
Difference from Elixhauser				-0.007 [-0.007,-0.006]	-0.009 [-0.009,-0.008]	0.011 [0.010,0.011]
**R^2: Outpatient log-cost**	0.093 [0.092,0.094]	0.088 [0.087,0.089]	0.156 [0.155,0.157]	0.095 [0.094,0.096]	0.318 [0.316,0.320]	0.364 [0.362,0.366]
Difference from Age-sex				0.002 [0.000,0.003]	0.225 [0.222,0.227]	0.271 [0.268,0.273]
Difference from Charlson				0.007 [0.005,0.008]	0.230 [0.228,0.231]	0.276 [0.274,0.278]
Difference from Elixhauser				-0.061 [-0.062,-0.059]	0.162 [0.160,0.164]	0.208 [0.206,0.210]

Note: Estimates represent medians across 1,000 bootstraps, with 95% confidence intervals (CI) derived from the central 95% of the bootstrap distribution. Reported R^2 values refer to explained variation on the log-cost scale. PopGroup results are based on all 753 PopGroups present in the study cohort.
