# Supplementary material for: Comparative predictive performance of the PopGrouper: internal validation of a population-based classification system for morbidity adjustment using German administrative data
Source: BMC Health Serv Res. 2026 Nov 12;26:1611. doi: 10.1186/s12913-026-16108-3 (PMC13770333; doi:10.1186/s12913-026-16108-3)
Supplement: Supplementary file 6 [file 12913_2026_16108_MOESM6_ESM.pdf]

Supplement F. Results of sensitivity analysis 3: Addition of age-sex groups to morbidity measure models

Performance metric: Outcome	Model				
	Charlson + Age-sex	Elixhauser + Age-sex	PopGroup severity + Age-sex	Macro PopGroup + Age-sex	PopGroup + Age-sex
**R^2: Total log-cost**	0.177	0.215	0.186	0.330	0.371
Difference from Charlson			0.009	0.153	0.194
Difference from Elixhauser			-0.029	0.115	0.156
**R^2: Inpatient log-cost**	0.078	0.092	0.087	0.082	0.100
Difference from Charlson			0.009	0.004	0.022
Difference from Elixhauser			-0.005	-0.010	0.008
**R^2: Outpatient log-cost**	0.148	0.191	0.153	0.335	0.370
Difference from Charlson			0.005	0.187	0.222
Difference from Elixhauser			-0.038	0.144	0.179

Performance metric: Outcome	Model				
	Charlson + Age-sex	Elixhauser + Age-sex	PopGroup severity + Age-sex	Macro PopGroup + Age-sex	Aggregated PopGroup + Age-sex
**c-statistic: Mortality**	0.902	0.924	0.924	0.923	0.932
Difference from Charlson			0.022	0.021	0.030
Difference from Elixhauser			0.000	-0.001	0.008
**c-statistic: Hospitalization**	0.701	0.715	0.712	0.714	0.726
Difference from Charlson			0.011	0.013	0.025
Difference from Elixhauser			-0.003	-0.001	0.010

Note: Models include age-sex groups in addition to the respective morbidity measure. Results are based on the full validation cohort without bootstrapping. R^2 values refer to the log-cost scale for cost outcomes based on all 753 PopGroups present in the study cohort. Aggregated PopGroups represent PopGroups or higher-level Base or Macro PopGroups with a minimum of 100 events per group (116 aggregated PopGroups for mortality and 449 for hospitalisation). For mortality, 15,069 individuals (MPG *P02*) were excluded.
