# Supplementary material for: Comparative predictive performance of the PopGrouper: internal validation of a population-based classification system for morbidity adjustment using German administrative data
Source: BMC Health Serv Res. 2026 Nov 12;26:1611. doi: 10.1186/s12913-026-16108-3 (PMC13770333; doi:10.1186/s12913-026-16108-3)
Supplement: Supplementary file 7 [file 12913_2026_16108_MOESM7_ESM.pdf]

Supplement G. Results of sensitivity analysis 4: Cross-classification of individuals in the top 10% predicted risk according to the Aggregated PopGroup and Elixhauser models

A) Mortality

**Risk group**	N	Observed mortality (%)	Mean predicted risk Elixhauser (%)	Mean predicted risk Aggregated PopGroup (%)
Both high-risk	70,926	38.7	31.3	31.3
Elixhauser only	58,641	10.3	17.4	3.8
Aggregated PopGroups only	56,589	14.0	3.4	22.3
Neither high-risk	1,109,048	1.1	1.7	1.8

Agreement: Cohen's κ = 0.502 (95% CI 0.500-0.505)

B) Hospitalisation

**Risk group**	N	Observed hospitalisation (%)	Mean predicted risk Elixhauser (%)	Mean predicted risk Aggregated PopGroup (%)
Both high-risk	67,124	42.4	42.2	37.3
Elixhauser only	63,903	31.9	36.6	23.4
Aggregated PopGroups only	66,781	30.8	12.4	35.9
Neither high-risk	1,112,465	12.0	12.4	12.4

Agreement: Cohen's κ = 0.451 (95% CI 0.449-0.454)

Note: High-risk was defined as belonging to the top 10% of predicted risk for the respective outcome according to each model. "Both high-risk" indicates individuals classified as high-risk by both models; "Elixhauser only" and "Aggregated PopGroup only" indicate individuals classified as high-risk by only one model. Cohen's κ quantifies agreement between the two high-risk classifications.
