# Supplementary material for: Comparative predictive performance of the PopGrouper: internal validation of a population-based classification system for morbidity adjustment using German administrative data
Source: BMC Health Serv Res. 2026 Nov 12;26:1611. doi: 10.1186/s12913-026-16108-3 (PMC13770333; doi:10.1186/s12913-026-16108-3)
Supplement: Supplementary file 8 [file 12913_2026_16108_MOESM8_ESM.pdf]

Supplement H. Results of sensitivity analysis 5: Agreement between observed and predicted outcomes

Supplement H-1. Agreement between observed and predicted outcomes across deciles of predicted total log-cost for Elixhauser and PopGroup models

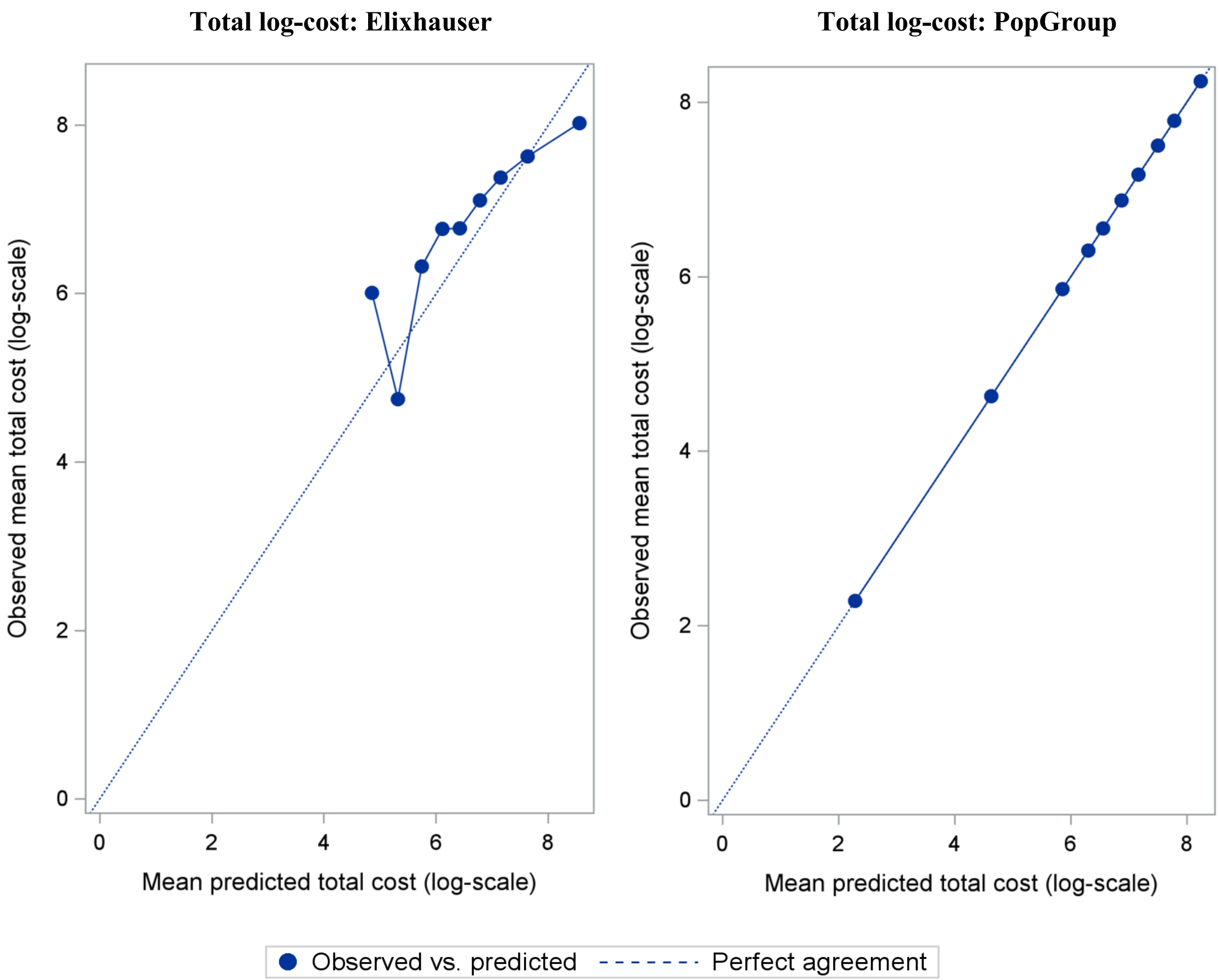

Note: Points represent deciles of predicted total log-cost. For each decile, the mean observed outcome is plotted against the mean predicted outcome. The dashed line indicates perfect agreement between observed and predicted values. Cost models were estimated using log-transformed costs; therefore, both axes are presented on the log-cost scale.

Supplement H-2. Agreement between observed and predicted outcomes across deciles of predicted inpatient log-cost for Elixhauser and PopGroup models

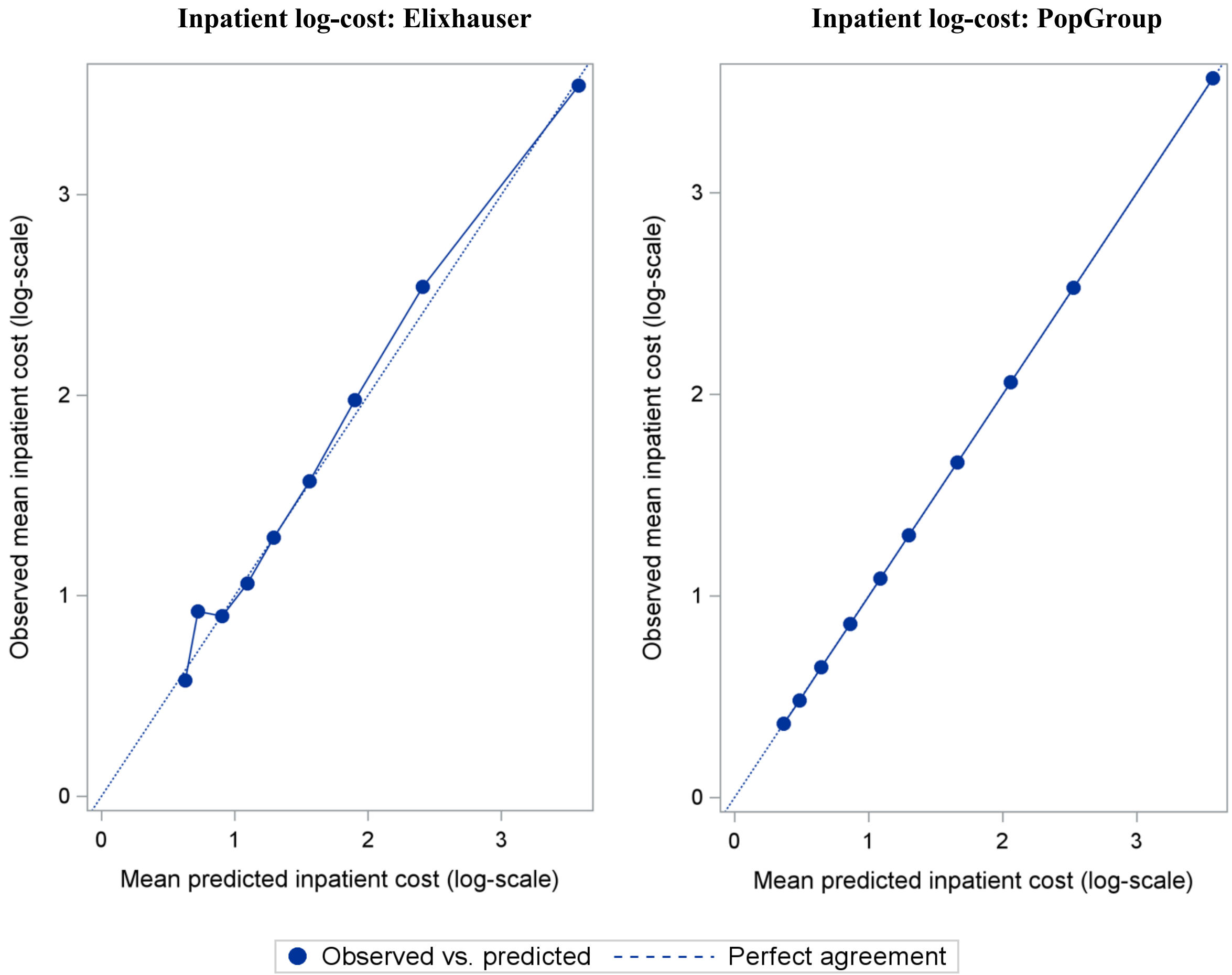

Note: Points represent deciles of predicted inpatient log-cost. For each decile, the mean observed outcome is plotted against the mean predicted outcome. The dashed line indicates perfect agreement between observed and predicted values. Cost models were estimated using log-transformed costs; therefore, both axes are presented on the log-cost scale.

Supplement H-3. Agreement between observed and predicted outcomes across deciles of predicted outpatient log-cost for Elixhauser and PopGroup models

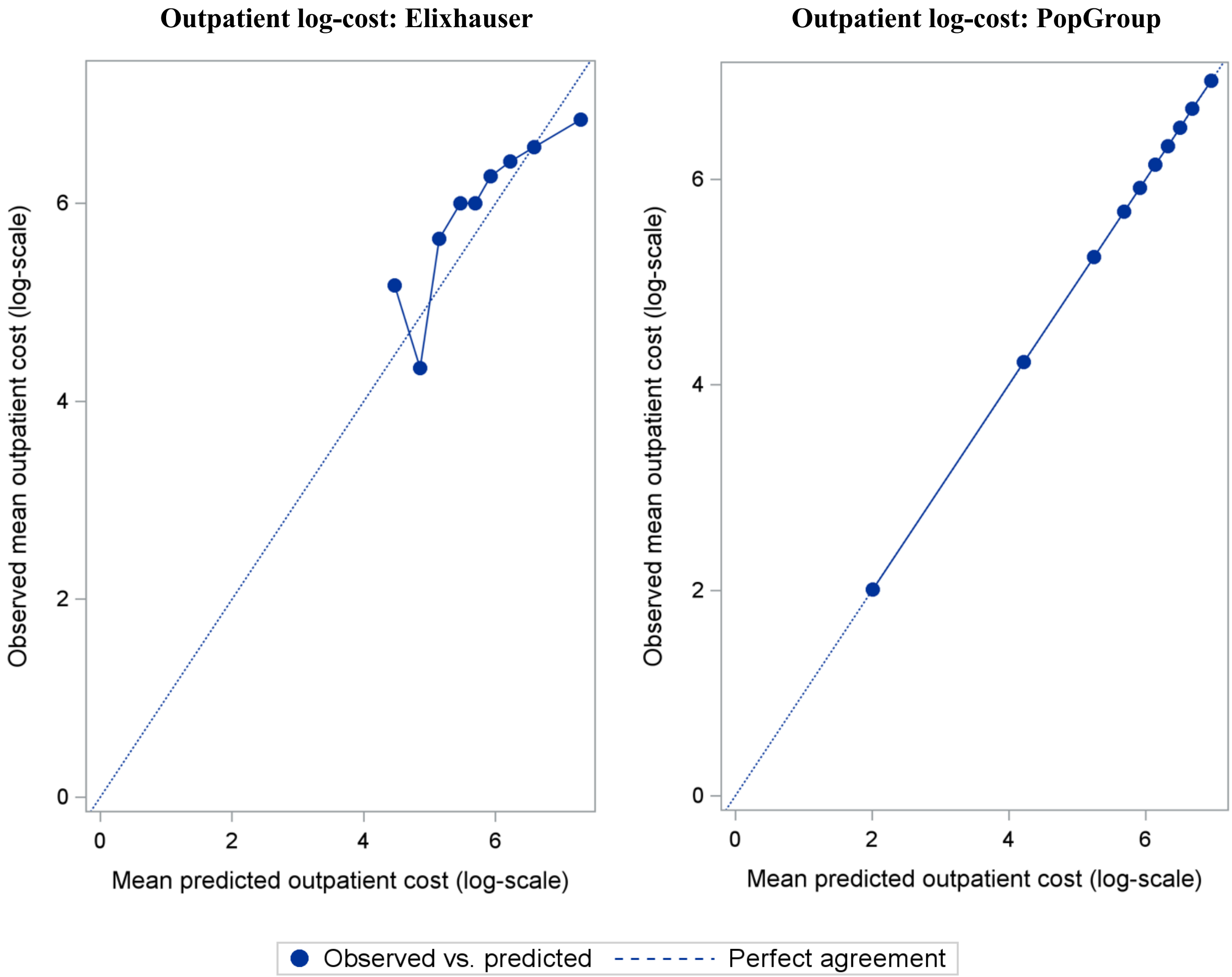

Note: Points represent deciles of predicted inpatient log-cost. For each decile, the mean observed outcome is plotted against the mean predicted outcome. The dashed line indicates perfect agreement between observed and predicted values. Cost models were estimated using log-transformed costs; therefore, both axes are presented on the log-cost scale.

Supplement H-4. Agreement between observed and predicted outcomes across deciles of predicted risk for Elixhauser and Aggregated PopGroup models

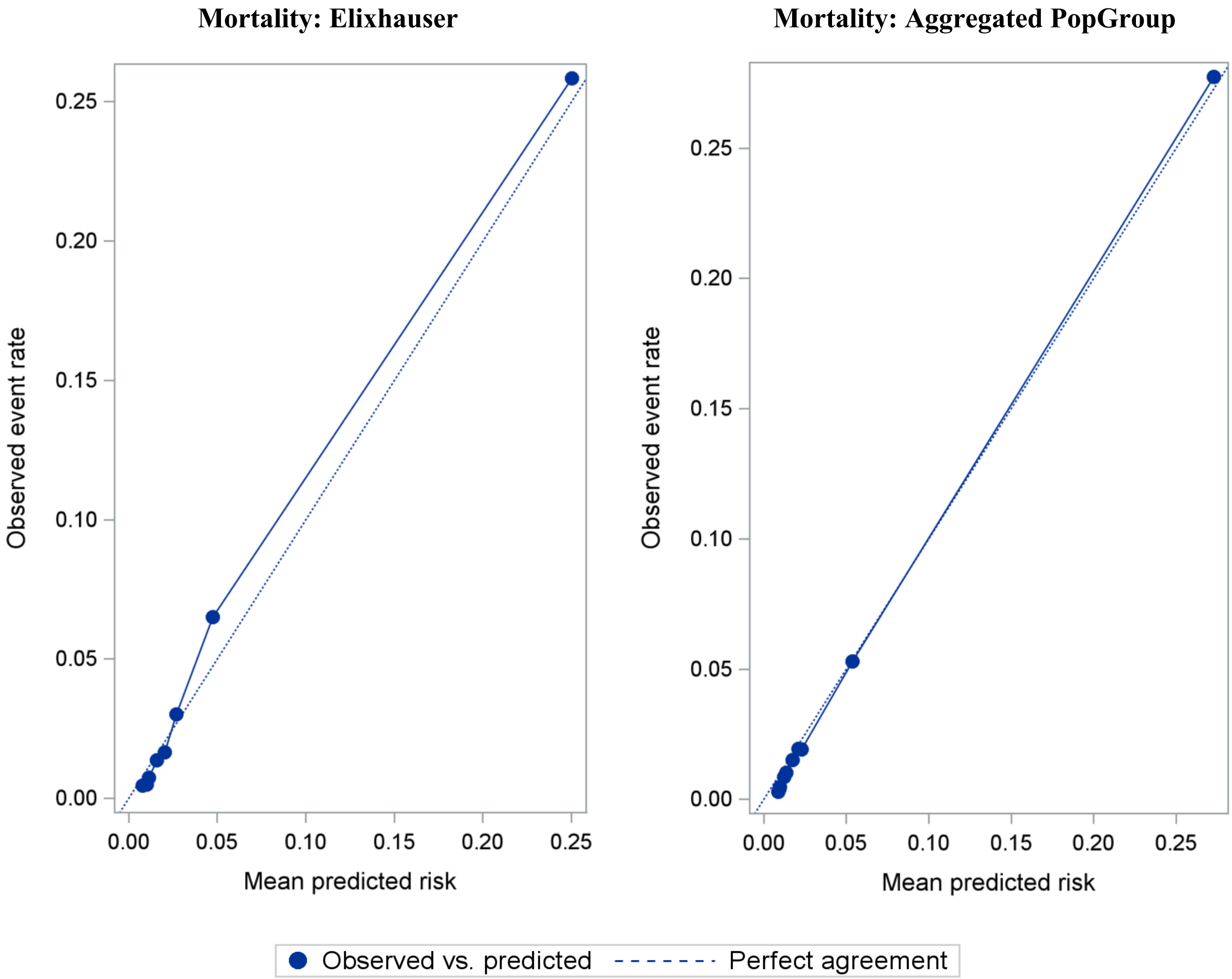

Note: Points represent deciles of predicted risk of 1-year mortality. For each decile, the mean observed risk is plotted against the mean predicted risk The dashed line indicates perfect agreement between observed and predicted values. Aggregated PopGroups represent PopGroups or higher-level Base or Macro PopGroups with a minimum of 100 events per group (116 aggregated PopGroups for mortality). For mortality, 15,069 individuals (MPG *P02*) were excluded.

Supplement H-5. Agreement between observed and predicted outcomes across deciles of predicted 1-year hospitalisation for Elixhauser and Aggregated PopGroup models

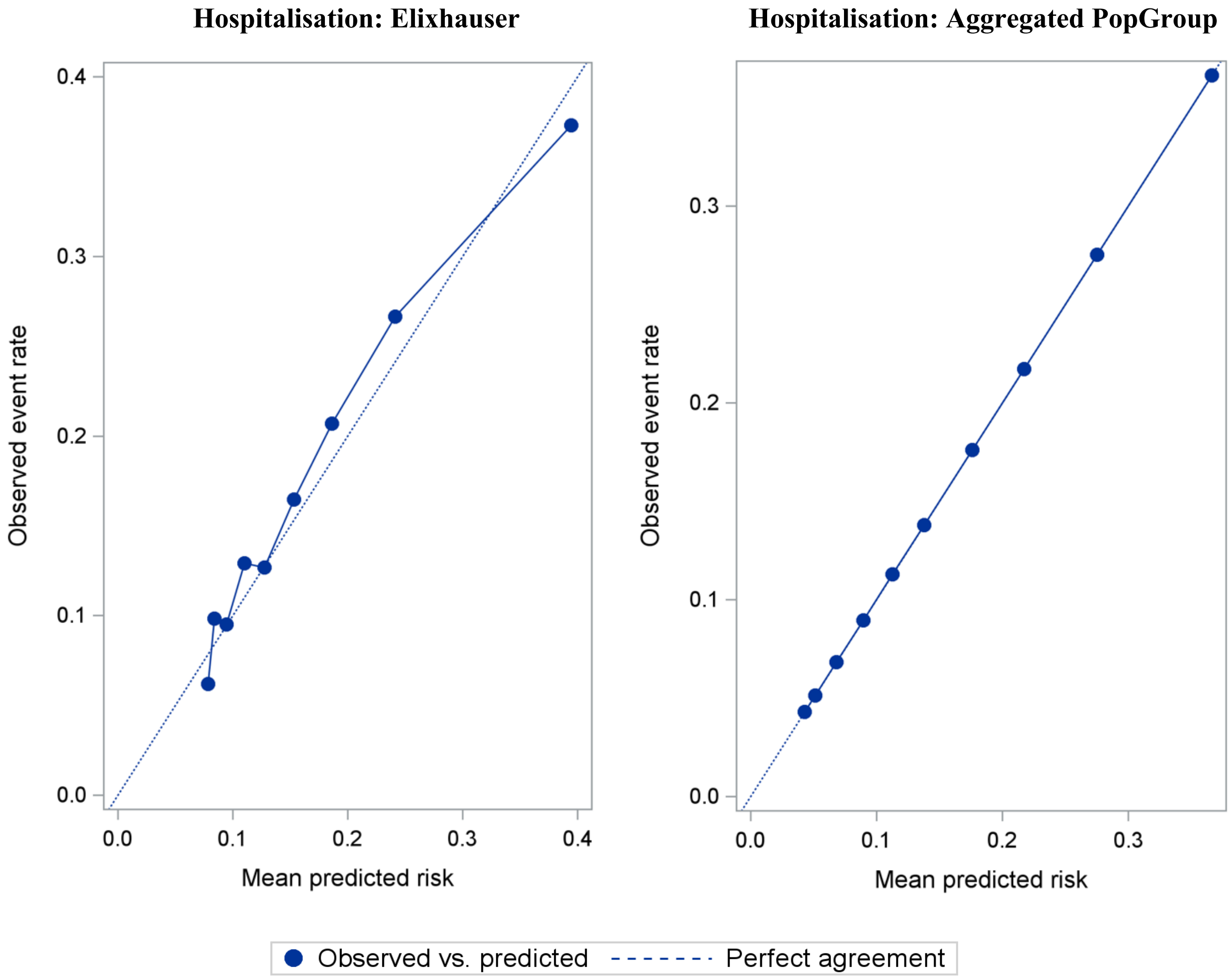

Note: Points represent deciles of predicted risk of 1-year hospitalisation. For each decile, the mean observed risk is plotted against the mean predicted risk The dashed line indicates perfect agreement between observed and predicted values. Aggregated PopGroups represent PopGroups or higher-level Base or Macro PopGroups with a minimum of 100 events per group (449 aggregated PopGroups for hospitalisation).
